# Quantitative spectral computed tomography detects different patterns of airway wall thickening and contrast enhancement in infective lung disease: a feasibility study

**DOI:** 10.1007/s00330-025-11752-5

**Published:** 2025-06-19

**Authors:** Philip Konietzke, Johanna Thomä, Oliver Weinheimer, Thuy D. Do, Willi L. Wagner, Arndt L. Bodenberger, Wolfram Stiller, Tim F. Weber, Claus P. Heußel, Hans-Ulrich Kauczor, Mark O. Wielpütz

**Affiliations:** 1 Diagnostic and Interventional Radiology (DIR), Heidelberg University Hospital (UKHD), Heidelberg, Germany; 2 Translational Lung Research Center Heidelberg (TLRC), German Center for Lung Research (DZL), Heidelberg, Germany; 3 Diagnostic and Interventional Radiology with Nuclear Medicine, Thoraxklinik at Heidelberg University Hospital (UKHD), Heidelberg, Germany

**Keywords:** Computed tomography, Lung, Inflammation, Pneumonia, COVID-19

## Abstract

**Objectives:**

We aimed to show that spectral computed tomography (CT) can identify different patterns of airway wall thickening and contrast enhancement in lung-healthy controls, coronavirus disease 2019 (COVID-19), and non-COVID-19 pneumonia patients, reflecting airway inflammation in both pneumonia subtypes and airway neovascularization in COVID-19.

**Materials and methods:**

331 subjects (age 58.9 ± 17.2 years) with 218 arterial and 113 venous phase spectral CT acquisitions were retrospectively recruited: 119 lung-healthy controls, 45 with COVID-19 and 167 with non-COVID-19 pneumonia. Scientific software was used for segmenting the airway tree. Wall thickness (WT_5-10_) and the difference in median maximum airway wall attenuation (slope of the spectral attenuation curve) between 40 keV and 100 keV display energy were calculated and aggregated for subsegmental airway generations 5–10 (λHU_5-10_). Descriptive statistics, correlations, *t*-tests, and ANOVA analyses were performed.

**Results:**

Arterial phase WT_5-10_ was similarly increased in COVID-19 (1.70 ± 0.44 mm) and non-COVID-19 (1.64 ± 0.53 mm) pneumonia compared to controls (1.18 ± 0.34 mm, *p* < 0.001). Arterial phase λHU_5-10_ was significantly higher in patients with COVID-19 pneumonia (3.09 ± 2.27 HU/keV) than in non-COVID-19 pneumonia (2.18 ± 1.54 HU/keV, *p* < 0.01) and lung-healthy controls (2.06 ± 1.11 HU/keV, *p* < 0.01).

**Conclusion:**

Spectral CT shows significant differences in segmental wall thickness and airway contrast enhancement between COVID-19 and non-COVID-19 pneumonia and lung-healthy controls. Airway contrast enhancement may be a feasible measure to detect airway inflammation in pneumonia and neovascularization in COVID-19 pneumonia.

**Key Points:**

***Question*** *Is spectral CT airway contrast enhancement a feasible quantitative method to detect airway inflammation or neovascularisation?*

***Findings*** *Spectral CT shows significant differences in segmental wall thickness and airway contrast enhancement between COVID-19 and non-COVID-19 pneumonia, and lung-healthy controls.*

***Clinical relevance*** *Spectral CT can be used to assess inflammatory airway diseases such as cystic fibrosis, COPD, asthma and bronchiectasis.*

**Graphical Abstract:**

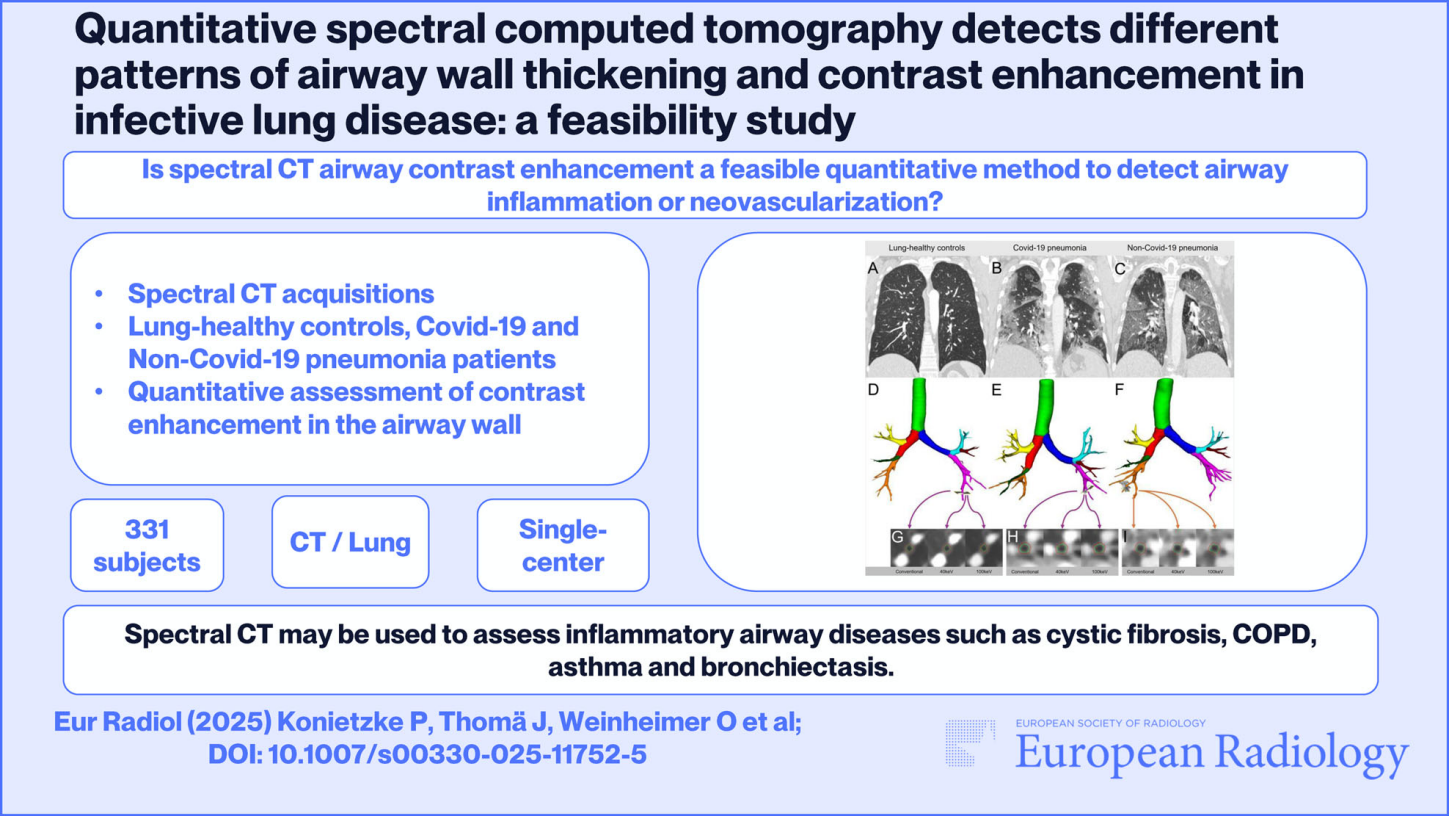

## Introduction

Quantitative post-processing of computed tomography (CT) imaging data is an increasingly used technique for the assessment of lung diseases, used mainly in airway diseases such as chronic obstructive pulmonary disease (COPD) and interstitial lung disease (ILD) [1–3]. Until 2019, the quantitative assessment of lung infections was not the focus of the research community, which changed with the emergence of the Coronavirus-19 (COVID-19) pandemic. In infective lung disease, alveolar infiltrates can cause an increase in lung density, while inflammatory bronchitis may cause thickening of the airway walls, which both can be detected with densitometry analyses and quantitative airway parameters [4, 5]. In the pathogenesis of COVID-19 pneumonia, the role of pulmonary hemodynamic changes is of great interest, and the thickening of the airway walls is partly attributed to expanded peribronchial vessels histologically [6, 7]. In this context, Lins et al also showed abnormalities in the distribution of blood volume within the pulmonary vascular tree, which is associated with increased pulmonary vascular resistance in the pulmonary vessels [8]. However, in vivo diagnostics of airway neovascularization have been precluded to date because of the limitations in spatial resolution of clinical CT. Airway wall contrast enhancement may be a surrogate for inflammation and neoangiogenesis, but was limited technically by multiphasic acquisitions and post-processing software required.

Dual-layer detector CT can selectively absorb low and high-energy photons simultaneously from the polychromatic X-ray spectrum of a single X-ray tube, thus enabling spectral imaging [9]. A previous study in lung-healthy controls demonstrated that airway wall contrast enhancement can be quantified from single-phase spectral CT by calculating the slope of the spectral attenuation curve based on Hounsfield Units (λHU) for lung parenchyma and airways based on virtual monochromatic imaging data sets at varying display energy levels [10].

In the present work, we aimed to show that parenchymal and airway spectral attenuation curves as quantitative spectral CT metrics can detect differences in wall thickening and airway wall contrast enhancement patterns in COVID-19, non-COVID-19 pneumonia and lung-healthy controls.

## Materials and methods

### Study subjects

**Fig. 1:**
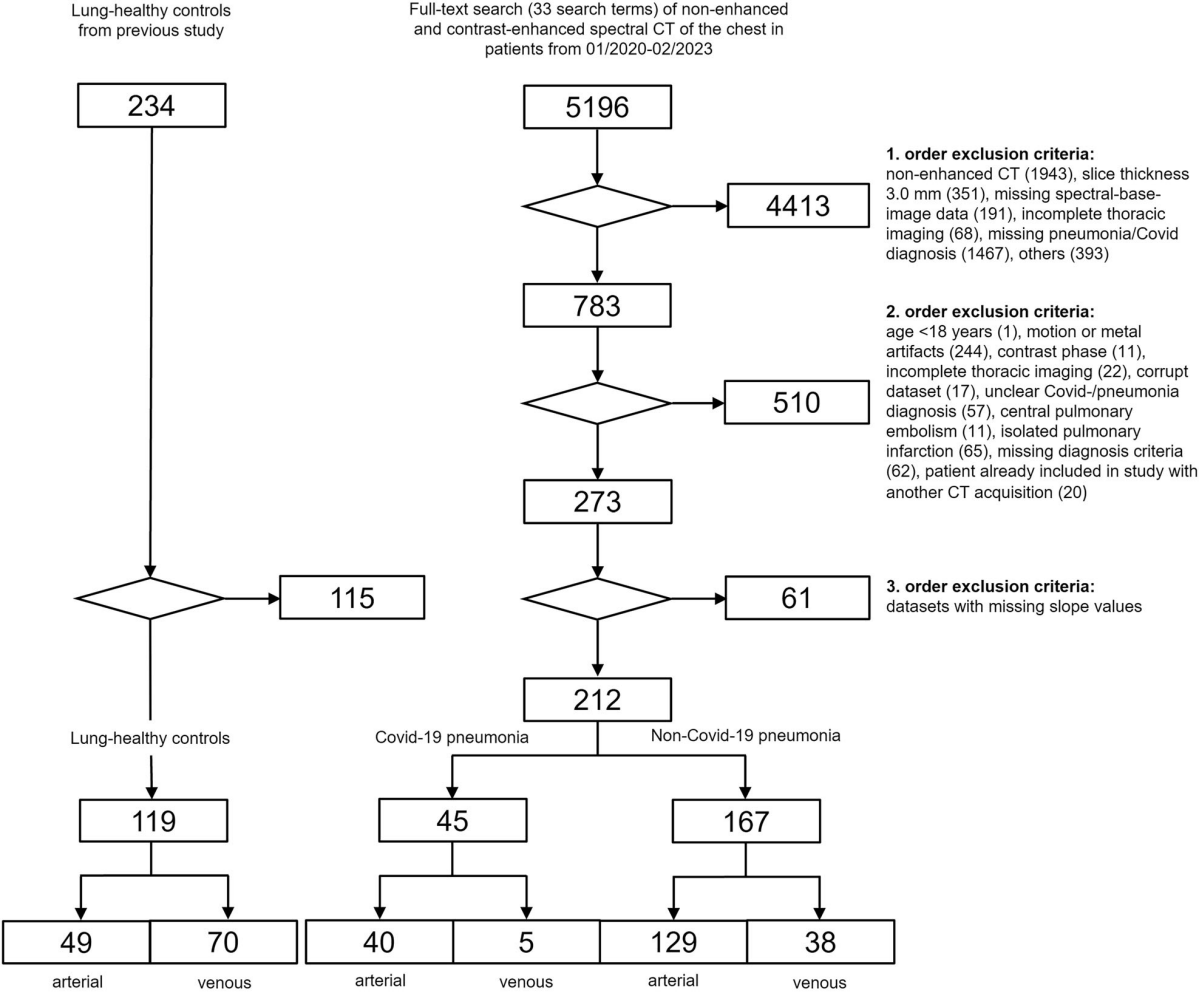
Subject recruitment flowchart. Inclusion and exclusion criteria for lung-healthy controls, see Bodenberger et al [10]

This retrospective single-center study was conducted in accordance with the Declaration of Helsinki. Ethical approval was granted by the local ethics committee of the Medical Faculty of Heidelberg University Hospital (S-924/2019), and the need for written informed consent was waived. A database search from 01/2020 to 02/2023 identified 5196 subjects who underwent clinically indicated contrast-enhanced spectral detector CT of the chest in arterial or venous phase. The clinical indications for CT were recorded and the first three suspected diagnoses mentioned in the CT request were divided into 5 groups: (1) pulmonary artery embolism (2) pneumonia (3) unknown focus of infection (4) oncological staging (5) other (all other indications, e.g., ischemia, hemorrhage, etc.). All subjects were reviewed for inclusion and exclusion criteria by two readers with one (J.T.) and fifteen (M.O.W.) years of experience in chest imaging. The inclusion criteria for non-COVID-19 pneumonia were (1) radiologic feature of consolidated lung, and/or (2) radiologic features of ground glass opacities consistent with infection, (3) age > 18, and (4) absence of severe motion artifacts. Detailed exclusion criteria are listed in the study flowchart (Fig. 1). Central pulmonary embolism was an exclusion criterion, whereas singular peripheral non-occlusive pulmonary embolism was tolerated. A total of 167 patients with non-COVID-19 pneumonia, of whom 129 patients had an arterial and 38 venous phase acquisitions, were recruited. 45 patients with COVID-19 pneumonia, with the additional criterion of a positive RT-PCR test, were recruited, of whom 40 patients had an arterial and 5 venous phase acquisition. In addition, 119 lung-healthy controls were included from a previously published patient collective [10]. Clinical information extracted from the hospital information system (I.S.-H.*med, SAP) included patient demographics (Table 1).

**Table 1:** Patient characteristics for lung-healthy controls, COVID-19 and non-COVID-19 pneumonia

	Lung-healthy controls	COVID-19 pneumonia	Non-COVID-19 pneumonia
Arterial phase			
Patient demographics			
*n*	49	40	129
Age	52.59 ± 17.38	61.78 ± 15.21*	61.53 ± 15.05*
Sex (m/f)	15/34	31/9	86/43
Height (cm)	170.30 ± 8.60	173.05 ± 7.65	173.13 ± 10.34
Weight (kg)	87.94 ± 26.62	79.61 ± 14.48	79.61 ± 14.48
BMI (kg/m²)	30.25 ± 8.78	26.52 ± 4.27*	26.22 ± 5.67*
Suspected clinical diagnosis			
PE	17 (35%)	34 (85%)	96 (75%)
Pneumonia	0 (0%)	18 (45%)	37 (29%)
Unknown infection	4 (9%)	7 (18%)	9 (7%)
Staging	23 (46%)	1 (3%)	10 (8%)
Other	5 (10%)	2 (5%)	44 (34%)
Imaging diagnosis			
Peripheral PE	0/49 (0%)	8/40 (20%)	16/129 (12.4%)
Venous phase			
Patient demographics			
*n*	70	5	38
Age	52.69 ± 17.64	58.60 ± 16.52	60.89 ± 16.54
Sex (m/f)	35/35	2/3	21/17
Height (cm)	171.19 ± 9.12	171.40 ± 5.20	171.43 ± 9.66
Weight (kg)	75.48 ± 19.48	73.60 ± 8.31	69.45 ± 14.87
BMI (kg/m²)	25.61 ± 5.55	25.09 ± 2.96	23.67 ± 4.95
Suspected clinical diagnosis			
PE	24 (35%)	0 (0%)	1 (3%)
Pneumonia	0 (0%)	1 (20%)	9 (24%)
Unknown infection	5 (7%)	0 (0%)	3 (8%)
Staging	36 (51%)	4 (80%)	25 (66%)
Other	7 (11%)	2 (40%)	13 (34%)
Imaging diagnosis			
Peripheral PE	0/70 (0%)	0/5 (0%)	3/38 (7.9%)

Patient demographics, clinical indications for CT and the prevalence of imaging diagnosis of peripheral, non-occlusive pulmonary embolism in lung-healthy controls, COVID-19 and non-COVID-19 pneumonia patients grouped by arterial and venous phase are shown. Data are given as mean ± standard deviation

*PE* pulmonary embolism, *BMI* body mass index

* *p* < 0.05 vs. healthy controls

### Spectral detector CT

Spectral detector CT of the chest was performed using a 64-row and a 128-row dual-layer CT with dose modulation in inspiratory breath hold (IQon Spectral CT and Spectral CT 7500; Philips Healthcare). Contrast material (Iohexol 350 mg iodine/mL, Accupaque 350, GE Healthcare) was applied by a power injector following standardized protocols (Table 2). The mean volumetric computed tomography dose index (CTDI_vol_) for arterial and venous phase acquisitions was 8.59 mGy and 5.99 mGy, respectively. Reconstructed slice thickness of 1.5 mm and increment of 0.75 mm were identical for all groups. Conventional imaging data reflecting 120 kVp and two computed virtual monogenetic imaging data sets (VMI) for 40 and 100 keV display energy were generated from spectral-base image data using software provided by the CT manufacturer (IntelliSpace Portal 11, Philips Healthcare). Please note that virtual monochromatic images are not measured but computed data.

**Table 2:** Spectral CT acquisition protocols and image reconstruction parameters in lung-healthy controls, COVID-19 and Non-COVID-19 pneumonia groups

	Arterial phase	Venous phase
Acquisition		
Collimation (mm)	64 × 0.625	128 × 0.625
Pitch	0.984	0.984
Rotation time (s)	0.33	0.33
kVp	120	120
mAs	102.81 ± 73.85	67.40 ± 58.87
CTDI_vol_ (mGy)	8.59 ± 6.55	5.99 ± 5.39
DLP (mGy*cm)	372.74 ± 267.71	231.92 ± 179.60
Contrast application		
Trigger region	Pulmonary trunk	Pulmonary trunk
Threshold (HU)	150	150
Delay (s)	6	35
Iodine (mg/mL)	350	350
Volume (mL)	56.94 ± 19.53	51.03 ± 8.63
Flow rate (mL/s)	1.5–4.5	2.0–4.5
Reconstruction		
Kernel	B	B
Spectral level	3	3
Image matrix	512 × 512	512 × 512
Slice thickness (mm)	1.5	1.5
Increment (mm)	0.75	0.75

Data are given as mean ± standard deviation

*DLP* dose length product, *CTDI*_*vol*_ volumetric computed tomography dose index

### Image analysis

To measure vascular enhancement, mean CT attenuation in Hounsfield Units (HU) was measured manually on conventional imaging data using a circular region-of-interest in the right pulmonary artery and the descending aorta with an area of 2.0 or 1.5 cm^2^ (± 0.05 cm^2^), and the inferior vena cava with an area of 1.5 or 1.0 cm^2^ (± 0.05 cm^2^).

**Fig. 2:**
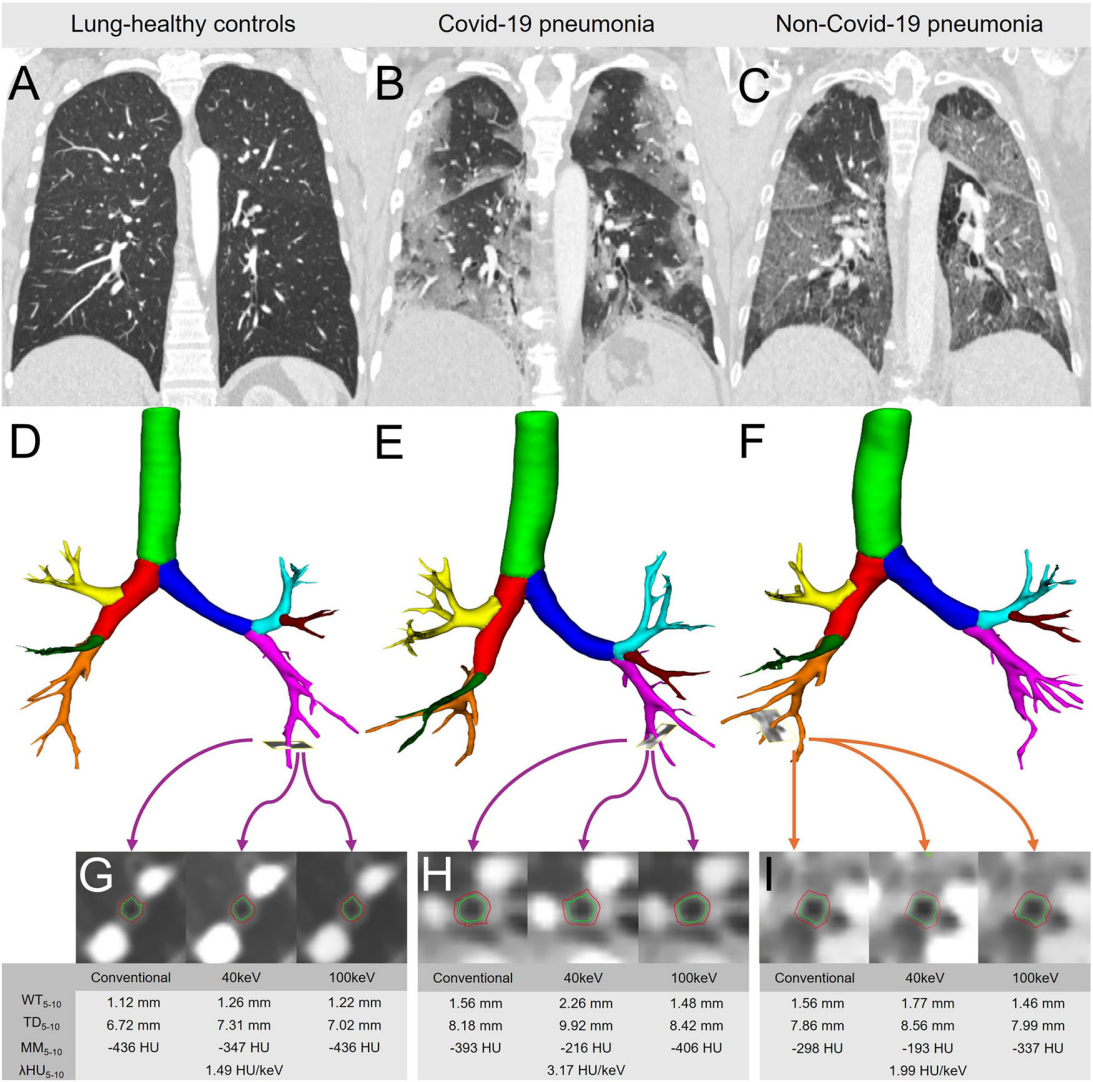
Quantified airway wall contrast enhancement in lung-healthy controls, COVID-19 and non-COVID-19 pneumonia. **A**–**C** Coronal CT images of lung-healthy controls and subjects with COVID-19 and non-COVID-19 pneumonia. In COVID-19 pneumonia, ground glass opacities showed a peripheral predominance. **D**–**F** Segmentation results of the airway tree. **G**–**I** Axial conventional, 40 keV and 100 keV CT image data of the segmented airway walls of a subsegmental bronchus (red = outer diameter, green = inner diameter)

The well-validated scientific software YACTA (version 2.9.1.12/16/31) segmented the lungs and the airway tree on conventional as well as on computed 40 keV and 100 keV display energy virtual monoenergetic imaging data sets as previously described [10–14]. Mean lung densities (MLD), representing the mean attenuation of all segmented lung voxels were quantified as previously described [15, 16]. Airway measurements were calculated using the modified integral-based-method, which removes airway wall segments attached to a vessel from calculations of all airway parameters, thus avoiding blooming of contrast enhancement into airway measurements as previously described [17]. The values for the 5–10th airway generation were aggregated as a pooled parameter for subsegmental airways, as done previously [10, 13, 18]. The Pi10 as standardized measure for WT was derived by plotting the square root of the airway wall area against the internal perimeter of the airway for every measured airway location [19]. The airway dimensions wall thickness (WT), representing the median distance between inner and outer airway wall border, and total diameter (TD), defined as the median distance from outer to outer border of the airway segment, were quantified as previously described [10, 11]. The median maximum airway wall attenuation (MM) in HU was recorded. λHU was calculated as the difference of 40–100 keV values for MLD and for MM from computed monoenergetic images by the formula ${{{\rm{\lambda }}}}{{{\rm{HU}}}}={{HU}}_{40{keV}}-{{HU}}_{100{keV}}/60 \, {keV}$ as described by Alvarez et al and used in oncological imaging [20–22]. Please note that the formula is a simplified linear approximation to the true exponential curve (Fig. 2).

### Statistical analysis

All data were recorded in a dedicated spreadsheet (Excel, Microsoft Corporation), and analyses were performed with SPSS (SPSS Statistics 27, IBM) and SigmaPlot (Systat Software GmbH). Data are given as mean ± standard deviation. Quantitative CT parameters within each specific contrast phase were analyzed using One-way analysis of variance (ANOVA), Tukey multiple pairwise comparisons (Tukey Test). Quantitative CT parameters between contrast phase groups were compared with Student’s *t*-test. Multiple linear regression was used to estimate the relationship between the independent variables and the dependent variable. A *p*-value < 0.05 was considered statistically significant.

## Results

### Contrast application and large vessel enhancement were comparable between study groups

To verify whether the level of vascular contrast enhancement was comparable between study groups, the CT number attenuation (HU) of the right pulmonary artery (RPA), descending aorta (DA), and inferior vena cava (VCI) were measured on conventional imaging data. In the arterial phase, mean CT number attenuation in Hounsfield Units (HU) in the DA was significantly higher in the lung-healthy group than in the COVID-19 and non-COVID-19 pneumonia (*p* = 0.004), while there were no significant differences in the RPA and VCI (*p* = 0.184, *p* = 0.906). In the venous phase, no significant differences were found between vessels and study groups (Table 3). Contrast application was compared between the lung-healthy group and the COVID-19 and non-COVID-19 pneumonia groups. In the arterial phase, there was no significant difference in contrast media volume, application duration and calculated flow rate between the study groups (*p* = 0.626, *p* = 0.266, *p* = 0.644). In the venous phase, contrast media volume was significantly higher in the lung-healthy group compared to the non-COVID-19 pneumonia groups (*p* = 0.050), but there was no significant difference between application duration and flow rate (*p* = 0.644, *p* = 0.260). Data were not available for the venous phase COVID-19 group (Table 3).

**Table 3:** Contrast application parameters and vessel opacification at spectral CT in lung-healthy controls, COVID-19 and non-COVID-19 pneumonia

	Lung-healthy controls	COVID-19 pneumonia	Non-COVID-19 pneumonia
Arterial phase			
CT number attenuation (HU)			
RPA	289 ± 91	291 ± 95	265 ± 101
DA	218 ± 72	158 ± 86*	180 ± 91*
VCI	45 ± 14	45 ± 8	45 ± 12
Contrast media application parameters			
Volume (mL)	60.6 ± 8.1	49.2 ± 24.6	56.6 ± 22.0
Duration (s)	17.8 ± 3.3	15.3 ± 8.2	17.2 ± 8.5
Flow rate (mL/s)	3.5 ± 3.5	3.3 ± 0.4	3.4 ± 0.5
Venous phase			
CT number attenuation (HU)			
RPA	116 ± 22	97 ± 23	102 ± 29
DA	119 ± 25	101 ± 23	103 ± 31
VCI	96 ± 28	77 ± 15	84 ± 25
Contrast media application parameters			
Volume (mL)	52.0 ± 7.0	N/A	47.7 ± 12.1*
Duration (s)	18.4 ± 3.0	N/A	16.9 ± 4.1
Flow rate (mL/s)	2.8 ± 0.2	N/A	2.9 ± 0.4

Mean CT number attenuation in Hounsfield Units (HU) of the right pulmonary artery (RPA), descending aorta (DA), and the vena cava inferior (VCI) and the parameters contrast media volume, application duration and calculated flow rate in the arterial and venous phase are shown. Data are given as mean ± standard deviation

* *p* < 0.05 vs. lung-healthy controls

A multilinear regression analysis was used to predict the dependent variables λHU_MLD_ and λHU_5-10_ using a linear combination of the independent variables (RPA, DA, VCI and contrast volume, application time and flow rate). In the arterial phase, RPA had a significant influence on λHU_MLD_ in the lung-healthy controls (*p* = 0.010) and on λHU_MLD_ and λHU_5-10_ in non-COVID-19 pneumonia (*p* = 0.006, *p* = 0.017) (Table 4).

**Table 4:** Multiple linear regression analysis

	Lung-healthy controls		COVID-19 pneumonia		Non-COVID-19 pneumonia	
	λHU_MLD_	λHU_5-10_	λHU_MLD_	λHU_5-10_	λHU_MLD_	λHU_5-10_
Arterial phase						
RPA (HU)	0.010	0.233	0.454	0.958	0.006	0.017
DA (HU)	0.818	0.962	0.370	0.333	0.084	0.348
VCI (HU)	0.296	0.661	0.114	0.603	0.861	0.553
Volume (mL)	0.277	0.821	0.191	0.241	0.782	0.826
Duration (s)	0.258	0.852	0.324	0.217	0.873	0.203
Flow rate (mL/s)	0.191	0.794	0.844	0.416	0.712	0.602
Venous phase						
RPA (HU)	0.504	0.118	0.366	0.508	0.060	0.063
DA (HU)	0.517	0.612	0.069	0.541	0.080	0.138
VCI (HU)	0.372	0.418	0.151	0.939	0.901	0.245
Volume (mL)	0.561	0.467	N/A	N/A	0.082	0.376
Duration (s)	0.730	0.486	N/A	N/A	0.068	0.361
Flow rate (mL/s)	0.749	0.453	N/A	N/A	0.070	0.320

Multiple linear regression was used to estimate the relationship between the independent variables (CT number attenuation in Hounsfield Units (HU) of the right pulmonary artery (RPA), descending aorta (DA), and the vena cava inferior (VCI) and the parameters contrast media volume, application duration and calculated flow rate) and a dependent variable (λHU_MLD_, λHU_5-10_) in the arterial and venous phase. A *p*-value < 0.05 was considered statistically significant

### Mean lung density is increased in COVID-19 and non-COVID-19 pneumonia

MLD was measured in both contrast phases on conventional imaging data to assess the influence of consolidation. In both contrast phases, MLD was significantly higher in the COVID**-**19 and non-COVID-19 pneumonia compared to the lung-healthy groups (both *p* < 0.001). In addition, in the venous phase, MLD was significantly lower in the COVID-19 than in the non-COVID-19 pneumonia group (*p* = 0.004) (Table 5).

**Table 5:** Quantitative CT parameters of the parenchyma and airways in lung-healthy controls, COVID-19 and non-COVID-19 pneumonia

	Lung-healthy controls	COVID-19 pneumonia	Non-COVID-19 pneumonia
Arterial phase			
MLD (HU)	−768 ± 34	−641 ± 72**	−657 ± 75**
Pi10 (mm)	0.28 ± 0.10	0.31 ± 0.11	0.35 ± 0.13*
WT_5-10_ (mm)	1.18 ± 0.34	1.70 ± 0.44**	1.64 ± 0.53**
TD_5-10_ (mm)	6.76 ± 0.80	8.32 ± 1.19**	8.05 ± 1.50**
MM_5-10_ (HU)	−436 ± 112	−303 ± 121**	−318 ± 147**
λHU_MLD_ (HU/keV)	1.28 ± 0.37	0.92 ± 0.70**	0.85 ± 0.43**
λHU_5-10_ (HU/keV)	2.06 ± 1.11	3.09 ± 2.27*^#^	2.18 ± 1.54
Venous phase			
MLD (HU)	−790 ± 33^§§^	−634 ± 49**^#^	−702 ± 61**^§§^
Pi10 (mm)	0.27 ± 0.08	0.42 ± 0.12**^#^	0.30 ± 0.10^§^
WT_5-10_ (mm)	1.15 ± 0.31	1.84 ± 0.31**^#^	1.39 ± 0.42*^§^
TD_5-10_ (mm)	6.76 ± 0.77	8.34 ± 0.62**^#^	7.23 ± 1.06*^§^
MM_5-10_ (HU)	−452 ± 113	−260 ± 109*^#^	−378 ± 133*^§^
λHU_MLD_ (HU/keV)	0.54 ± 0.14^§§^	0.60 ± 0.21	0.48 ± 0.46^§§^
λHU_5-10_ (HU/keV)	0.90 ± 1.10^§§^	1.29 ± 1.36	0.88 ± 1.62^§§^

Kindly note that quantitative parameters are derived from conventional imaging data, and the slope of the spectral attenuation curve from virtual monoenergetic imaging data sets at 40 and 100 keV display energy

* *p* < 0.05 vs. lung-healthy controls

** *p* < 0.01 vs. lung-healthy controls

# *p* < 0.05 vs. Non-COVID-19 pneumonia

## *p* < 0.01 vs. Non-COVID-19 pneumonia

§ *p* < 0.05 vs. same group in the arterial phase

§§ *p* < 0.01 vs. same group in the arterial phase

### Airway wall thickening is similar in COVID-19 and non-COVID-19 pneumonia

Airway dimensions were assessed on conventional imaging data in arterial and venous contrast phases to study the effects of inflammatory wall thickening. Between the arterial and venous phases, the airway parameters Pi10, WT_5-10_, TD_5-10_ and MM_5-10_ showed no significant differences in the lung-healthy controls and COVID-19 pneumonia. As an exception, all parameters in Non-COVID-19 pneumonia were significantly higher in the arterial phase than in the venous phase (all *p* < 0.005) (Table 5). In the arterial phase, Pi10 was significantly higher in Non-COVID-19 and tended to be higher in the COVID-19 subtype than in the lung-healthy controls (*p* = 0.002 and *p* = 0.310, respectively), while there was no significant difference between the pneumonia subtypes (*p* = 0.303). In the venous phase, Pi10 was significantly higher in COVID-19 than in non-COVID-19 pneumonia and lung-healthy controls (*p* < 0.001, *p* = 0.012). WT_5-10_ and TD_5-10_ were significantly higher in both pneumonia subtypes than in lung-healthy controls in both contrast phases (*p* < 0.001). In the arterial phase, WT_5-10_ and TD_5-10_ showed no differences between pneumonia subtypes, while in the venous phase, both parameters were significantly higher in COVID-19 than in non-COVID-19 pneumonia (*p* < 0.05). MM_5-10_ was higher in both pneumonia subtypes than in lung-healthy controls in both contrast phases (*p* < 0.001). In the arterial phase, there were no differences between the pneumonia subtypes, while in the venous phase, MM_5-10_ was significantly higher in the COVID-19 subtype than in the non-COVID-19 subtype (*p* < 0.001) (Table 5).

### Reduced parenchymal contrast enhancement in COVID-19 and non-COVID-19 pneumonia

The slope of the spectral attenuation curve λHU_MLD_ based on the imaging data sets at 40 and 100 keV display energy was higher in the arterial phase than in the venous phase within lung-healthy controls and non-COVID-19 pneumonia (all *p* < 0.001), but not in COVID-19 pneumonia (*p* = 0.339). In the arterial phase, λHU_MLD_ was significantly higher in the lung-healthy group than in both pneumonia subtypes (*p* < 0.001), while there were no differences between the groups in the venous phase (Table 5).

### Airway wall contrast enhancement differentiates COVID-19 from non-COVID-19 pneumonia

To quantify airway wall enhancement, we then calculated the slope of the spectral attenuation curve λHU for the airways aggregated for the 5–10th generation. λHU_5-10_ was significantly higher in the arterial than in the venous phase in lung-healthy controls and non-COVID-19 pneumonia (all *p* < 0.001), but not in COVID-19 pneumonia (*p* = 0.049). In the arterial phase, λHU_5-10_ was higher in COVID-19 pneumonia than in non-COVID-19 pneumonia and lung-healthy controls (*p* = 0.009 and *p* = 0.004, respectively), while there was no difference between non-COVID-19 pneumonia and controls (*p* = 0.907) (Table 5). Further, no differences in the venous phase were observed.

## Discussion

With previous work, we showed that λHU could be a suitable parameter to assess lung parenchyma and airway wall contrast enhancement from single-phase spectral CT. In this work, we demonstrate for the first time that spectral CT can detect airway wall enhancement by distinguishing between COVID-19 pneumonia patients, non-COVID-19 pneumonia patients and lung-healthy controls based on an increased arterial-phase airway wall contrast enhancement, whereas both COVID-19 and non-COVID-19 pneumonia demonstrate quantitatively comparable thickened airway walls.

The contrast phase plays a key role in the interpretation of our results. In normal subjects, the flow of intravenous contrast through an upper extremity venous access is first into the brachiocephalic vein, then into the superior vena cava, right atrium, right ventricle, pulmonary artery, pulmonary vein, left atrium, left ventricle, and finally into the ascending and then the descending aorta. The bronchial arteries are vascular branches that arise from the descending aorta and supply the bronchial walls with arterial blood. At the end of the circulation cycle, the contrast media reenters the vena cava and from there the right atrium [23]. In the arterial phase, the lung parenchyma should have its first-pass peak enhancement as the bolus passes through the pulmonary capillaries from the pulmonary artery into the pulmonary vein, whereas the first-pass enhancement of the airway walls should occur slightly later, as the bolus passes through the descending aorta supplying the bronchial arteries. In the venous phase, the bolus is increasingly *“stretched”* throughout the vascular system, making contrast enhancement more homogeneous. Therefore, we expected that opacification of the lung parenchyma should be associated mainly with contrast enhancement of the pulmonary arteries, whereas enhancement of the airway wall should be associated mainly with contrast enhancement of the thoracic aorta.

To assess whether contrast phase was comparable between study groups, the mean contrast opacification was measured in the right pulmonary artery (RPA), the descending aorta (DA) and the inferior vena cava (VCI). Our results show that in the arterial phase, the mean CT number in the aorta was significantly higher in the lung-healthy control group than in the COVID-19 and non-COVID-19 pneumonia patient groups, whereas in the venous phase, no significant differences were found between vessels and study groups. The literature reports that the optimal contrast enhancement for the pulmonary arteries is 250–300 HU, while experimental models suggest that adequate arterial opacification in aortal CTA protocols is > 200 HU [24–26]. In our study, all three groups reached the desired 250–300 HU in the RPA, while the desired > 200 HU in the aorta was only reached in lung-healthy controls (Table 3). In addition, arterial peak enhancement should also be considered, as already discussed for abdominal organs [27]. In the abdominal arterial phase CT scans are usually divided into an early arterial phase, corresponding to contrast arrival in the aorta, where only vessels are opacified (15–20 s after injection or immediately after bolus tracking), and a late arterial phase, where highly perfused tissues show their maximum contrast enhancement (35–40 s after injection or 15–20 s after bolus tracking) [25, 28, 29]. Cressoni et al showed that peak tissue enhancement occurs at the end of the contrast bolus transit, which represents the optimal time for abdominal arterial phase scanning [27]. Considering that airway wall enhancement should occur as the bolus passes through the descending aorta by supplying the bronchial arteries, and translating the observations from abdominal to airway imaging, a slightly longer delay time of 10–12 s is likely to be more favorable for achieving optimal airway wall enhancement.

We also evaluated contrast application parameters for potential differences between all three study groups, as injection duration and rate affected aortic peak time and peak enhancement [30]. In the arterial phase, there was no significant difference in contrast volume, application duration and calculated flow rate, whereas in the venous phase, contrast volume was significantly higher in the lung-healthy group compared with the non-COVID-19 pneumonia groups. The contrast volume was slightly higher in the lung-healthy group, probably due to the significantly higher BMI of the group, as contrast volume was routinely adjusted for weight. Overall, considering the contrast phases and contrast media application, we believe that our data are sufficient for an initial study to assess airway wall enhancement.

A multilinear regression analysis was used to predict the dependent variables λHU_MLD_ and λHU_5-10_ using a linear combination of the independent variables (RPA, DA, VCI and contrast volume, application time and flow rate) (Table 4). In the arterial phase, the attenuation of the RPA had a significant effect on λHU_MLD_ in the lung-healthy controls and non-COVID-19 pneumonia, which is in line with expectations, as contrast uptake of the lung parenchyma should be influenced by the pulmonary arteries. However, this correlation was not significant in the COVID-19 pneumonia group. It was also expected that the attenuation in DA would significantly influence λHU_5-10_ in all groups, but this was only the case in the non-COVID-19 pneumonia group. In conclusion, the expected correlations did not occur in all groups, and further studies with larger patient groups are needed to verify these results. However, the contrast media administration parameters did not seem to have an effect (Table 4).

The CT number in HU is a quantitative measurement of X-ray attenuation and the most frequently used quantitative CT parameter. In our study, the mean lung density in both contrast phases was significantly higher in COVID-19 and non-COVID-19 pneumonia patients than in lung-healthy controls. This was in line with expectations, as lung density increases in pneumonia due to complete or partial filling of air spaces, interstitial thickening, partial collapse of the alveoli, increased capillary blood volume or a combination of these factors [31]. In this context, the specific influence of capillary blood volume must be discussed, as contrast enhancement is influenced by the number of capillaries per voxel and the complex interaction of pathophysiological mechanisms that regulate local perfusion. Pneumonia causes regional alveolar hypoxia, which leads to hypoxic pulmonary vasoconstriction and reduces pulmonary blood flow, while simultaneously active inflammation in pneumonia can increase blood flow [32]. In our study, the arterial phase λHU_MLD_ was significantly lower in COVID-19 pneumonia patients and non-COVID-19 pneumonia patients than in lung-healthy controls, indicating an overall reduced perfusion of the lung parenchyma represented by small pulmonary vessels below the spatial resolution limits of clinical CT scanners. In accordance, Lins et al found reduced pulmonary blood volume in small vessels with a diameter < 5 mm in COVID-19 pneumonia patients [8]. The arterial phase λHU_MLD_ could also help to differentiate pneumonia from atelectasis, as it is commonly known that consolidations show less contrast uptake than atelectasis at contrast-enhanced CT. This is also in line with previous work using CT to differentiate atelectasis from pneumonic consolidations [33, 34]. Importantly, λHU_MLD_ did not differ between pneumonia subtypes and showed no significant differences in the venous phase between all groups, indicating that this parameter is indeed linked to perfusion.

Thickening of the bronchial walls may represent various histological changes such as edema, active inflammation, chronic remodeling, or thin layers of mucus [14, 19, 35, 36]. In the arterial and venous phases, Pi10 tended to be higher in both COVID-19 and non-COVID-19 pneumonia subtypes than in the lung-healthy control group. However, the differences to the lung-healthy control group were only significant for non-COVID-19 pneumonia in the arterial phase and for COVID-19 pneumonia in the venous phase. However, aggregated airway wall thickness and total diameter for subsegmental airways 5–10th generation were significantly increased in both COVID-19 and non-COVID-19 pneumonia, compared to lung-healthy controls in both contrast phases, while a difference between COVID-19 and non-COVID-19 pneumonia was only significant in the venous phase. Median maximum airway attenuation was significantly higher in both pneumonia subtypes than in lung-healthy controls in arterial and venous contrast phases (Table 5). Based on previous work, median maximum airway wall attenuation correlates well with airway wall thickness, which explains the similar behavior [37]. However, median maximum airway attenuation (MM) does not reflect airway enhancement sufficiently, as baseline attenuation without iodine is not captured. This was corrected by employing λHU_5-10_ to assess the contribution of iodine in the quantification. In summary, quantitative airway parameters allowed the differentiation between pneumonia and lung-healthy controls by detecting thickening of the subsegmental airways.

In COVID-19 pneumonia, the microvascular architecture of the peribronchial vessels is densely packed, showing distinctly dilated aberrant bundles of blood vessels, which are mainly supplied by bronchial arteries. This was demonstrated using micro-computed tomographic imaging, scanning electron microscopy and corrosion casting in postmortal specimens [6]. In the present work, our data show significantly higher λHU_5-10_ for COVID-19 pneumonia in the arterial phase than in non-COVID-19 pneumonia as well as in lung-healthy controls, while there was no difference between the non-COVID-19 subtype and lung-healthy controls (Table 5). We believe that λHU_5-10_ may successfully depict the increased arterial blood flow in the airway walls caused by COVID-19. This assumption is also supported by the differences in contrast phase, with the measured CT number in the descending aorta being significantly higher in the lung-healthy controls than in the pneumonia groups, indicating theoretically greater contrast enhancement of the bronchial arteries and thus a higher λHU_5-10_ in the lung-healthy controls. In the venous phase, λHU_5-10_ tended to be higher in COVID-19 pneumonia than in the other two groups, suggesting that the airway perfusion differences may occur predominantly in the early phase of the pulmonary circulation, which is in line with an increased bronchial arterial supply. A potential delayed enhancement pattern in the venous phase could not be found, which may be partly due to a limited population with venous phase acquisitions.

This work has the following limitations. (1) Contrast phase and enhancement are strongly influenced by extrinsic factors such as injection technique/equipment failure (IV cannula, power injector) and intrinsic patient-related factors (BMI and cardiac output) [23, 30, 38]. In this study, we attempted to control the influence of extrinsic factors by evaluating vessel enhancement and contrast administration parameters, but due to the nature of a retrospective setting with standard clinical protocols, the ability to adjust CT protocols for optimal airway wall enhancement was not possible. The influence of intrinsic factors was reduced as contrast volume was routinely adjusted for weight. However, BMI and cardiac output were not specifically considered in the data analysis. Overall, it cannot be excluded that extrinsic and intrinsic factors influenced the results, but we believe that this study can still serve as proof of concept, as the results are plausible. Future studies are needed to gain a deeper understanding of airway wall contrast enhancement. (2) The non-COVID-19 pneumonia group was very heterogeneous, as it was not subdivided into other pathogen classes (e.g., viruses, fungi, etc.) to assess whether arterial enhancement remained higher in COVID-19 patients compared to other specific pneumonia types. However, as the aim of this study was to show that spectral CT can detect differences in airway contrast enhancement, we believe that further subdivision of the patient groups would not necessarily have been beneficial. Nevertheless, future studies should investigate whether the observed pattern of enhancement is specific to COVID-19, as it may also occur in other viral pneumonias. Another major shortcoming in this context is the lack of histological validation of neovascularization in COVID-19, as the presumed associations are based solely on reports from the literature. (3) The prevalence of peripheral non-occlusive pulmonary embolism (PE) in pneumonia ranged from 7.9% to 20%, whereas no PE was diagnosed with CT in lung-healthy controls. We believe that this is acceptable, as peripheral, non-occlusive PE is unlikely to be associated with significant perfusion defects. We believe that the advantage of a larger number of patients in the study outweighs the disadvantage of a possible, probably small, influence on the results. Another aspect is the strong association of COVID-19 with microvascular thrombosis, the possible influence of which cannot be determined in this study [39]. (4) Conventional image data reflecting 120 kVp and two computed virtual monogenetic images for 40 and 100 keV display energy were used in this study. The formula used to calculate the contrast uptake assumes a linear relationship between the 40 and 100 keV images and the measured parameters, which is a simplistic approach, as a non-linear relationship is expected. This might influence our results and should be further investigated in future studies.

In conclusion, our data showed thickened subsegmental airway walls in COVID-19 and non-COVID-19 pneumonia, but different airway wall contrast enhancement patterns, which may represent pathological peribronchial neoangiogenesis and hyperemia in COVID-19 pneumonia. Therefore, we demonstrated that the slope of the arterial contrast phase spectral attenuation curve could be a feasible method to detect airway wall contrast enhancement. Although this was a promising first step, future studies should assess its applicability to other inflammatory airway diseases such as cystic fibrosis, COPD, asthma and bronchiectasis.

## Abbreviations

COVID-19: Coronavirus disease 2019
MLD: Mean lung density
MM: Median maximum airway wall attenuation
TD: Total diameter
WT: Wall thickness
λHU: Slope of the spectral attenuation curve

## References

[1] Iwasawa T, Matsushita S, Hirayama M, Baba T, Ogura T (2023) Quantitative analysis for lung disease on thin-section CT. Diagnostics (Basel) 13:2988 37761355 10.3390/diagnostics13182988 PMC10528918

[2] Mohamed Hoesein FA, de Hoop B, Zanen P et al (2011) CT-quantified emphysema in male heavy smokers: association with lung function decline. Thorax 66:782–787 21474499 10.1136/thx.2010.145995

[3] Bernstein EJ, Barr RG, Austin JHM et al (2016) Rheumatoid arthritis-associated autoantibodies and subclinical interstitial lung disease: the multi-ethnic study of atherosclerosis. Thorax 71:1082–1090 27609750 10.1136/thoraxjnl-2016-208932 PMC5342945

[4] Colombi D, Bodini FC, Petrini M et al (2020) Well-aerated lung on admitting chest CT to predict adverse outcome in COVID-19 pneumonia. Radiology 296:E86–E96 32301647 10.1148/radiol.2020201433 PMC7233411

[5] Sun Z, Liu X, Wang J, Wang X, Liu W, Chen Y (2020) Computed tomography evaluation of airway changes in adult patients with COVID-19 pneumonia. J Coll Physicians Surg Pak 30:785–789 32893786 10.29271/jcpsp.2020.08.785

[6] Ackermann M, Tafforeau P, Wagner WL et al (2022) The bronchial circulation in COVID-19 pneumonia. Am J Respir Crit Care Med 205:121–125 34734553 10.1164/rccm.202103-0594IM PMC8865596

[7] Hashemi-Madani N, Emami Z, Janani L, Khamseh ME (2021) Typical chest CT features can determine the severity of COVID-19: a systematic review and meta-analysis of the observational studies. Clin Imaging 74:67–75 33444992 10.1016/j.clinimag.2020.12.037 PMC7837254

[8] Lins M, Vandevenne J, Thillai M et al (2020) Assessment of small pulmonary blood vessels in COVID-19 patients using HRCT. Acad Radiol 27:1449–1455 32741657 10.1016/j.acra.2020.07.019 PMC7381940

[9] McCollough CH, Leng S, Yu L, Fletcher JG (2015) Dual- and multi-energy CT: principles, technical approaches, and clinical applications. Radiology 276:637–653 26302388 10.1148/radiol.2015142631 PMC4557396

[10] Bodenberger AL, Konietzke P, Weinheimer O et al (2023) Quantification of airway wall contrast enhancement on virtual monoenergetic images from spectral computed tomography. Eur Radiol 33:5557–5567 36892642 10.1007/s00330-023-09514-2 PMC10326154

[11] Weinheimer O, Achenbach T, Bletz C, Duber C, Kauczor HU, Heussel CP (2008) About objective 3-D analysis of airway geometry in computerized tomography. IEEE Trans Med Imaging 27:64–74 18270063 10.1109/TMI.2007.902798

[12] Weinheimer O, Achenbach T, Düber C (2009) Fully automated extraction of airways from CT scans based on self-adapting region growing. In: Proceedings of second international workshop on pulmonary image analysis, London

[13] Konietzke P, Weinheimer O, Wielputz MO et al (2018) Quantitative CT detects changes in airway dimensions and air-trapping after bronchial thermoplasty for severe asthma. Eur J Radiol 107:33–38 30292270 10.1016/j.ejrad.2018.08.007

[14] Wielputz MO, Eichinger M, Weinheimer O et al (2013) Automatic airway analysis on multidetector computed tomography in cystic fibrosis: correlation with pulmonary function testing. J Thorac Imaging 28:104–113 23222199 10.1097/RTI.0b013e3182765785

[15] Heussel CP, Kappes J, Hantusch R et al (2010) Contrast enhanced CT-scans are not comparable to non-enhanced scans in emphysema quantification. Eur J Radiol 74:473–478 19376661 10.1016/j.ejrad.2009.03.023

[16] Jobst BJ, Weinheimer O, Trauth M et al (2018) Effect of smoking cessation on quantitative computed tomography in smokers at risk in a lung cancer screening population. Eur Radiol 28:807–815 28884215 10.1007/s00330-017-5030-6

[17] Konietzke P, Weinheimer O, Wagner WL et al (2020) Optimizing airway wall segmentation and quantification by reducing the influence of adjacent vessels and intravascular contrast material with a modified integral-based algorithm in quantitative computed tomography. PLoS One 15:e0237939 32813730 10.1371/journal.pone.0237939 PMC7437894

[18] Konietzke P, Wielpütz MO, Wagner WL et al (2020) Quantitative CT detects progression in COPD patients with severe emphysema in a 3-month interval. Eur Radiol 30:2502–2512 31965260 10.1007/s00330-019-06577-y

[19] Grydeland TB, Dirksen A, Coxson HO et al (2009) Quantitative computed tomography: emphysema and airway wall thickness by sex, age and smoking. Eur Respir J 34:858–865 19324952 10.1183/09031936.00167908

[20] Alvarez RE, Macovski A (1976) Energy-selective reconstructions in X-ray computerized tomography. Phys Med Biol 21:733–744 967922 10.1088/0031-9155/21/5/002

[21] Kim C, Kim W, Park SJ et al (2020) Application of dual-energy spectral computed tomography to thoracic oncology imaging. Korean J Radiol 21:838–850 32524784 10.3348/kjr.2019.0711 PMC7289700

[22] Jia Y, Xiao X, Sun Q, Jiang H (2018) CT spectral parameters and serum tumour markers to differentiate histological types of cancer histology. Clin Radiol 73:1033–1040 30115364 10.1016/j.crad.2018.07.104

[23] Chaturvedi A, Oppenheimer D, Rajiah P, Kaproth-Joslin KA, Chaturvedi A (2017) Contrast opacification on thoracic CT angiography: challenges and solutions. Insights Imaging 8:127–140 27858323 10.1007/s13244-016-0524-3 PMC5265191

[24] Bae KT (2010) Optimization of contrast enhancement in thoracic MDCT. Radiol Clin North Am 48:9–29 19995627 10.1016/j.rcl.2009.08.012

[25] Bae KT (2010) Intravenous contrast medium administration and scan timing at CT: considerations and approaches. Radiology 256:32–61 20574084 10.1148/radiol.10090908

[26] Murphy DJ, Aghayev A, Steigner ML (2018) Vascular CT and MRI: a practical guide to imaging protocols. Insights Imaging 9:215–236 29541955 10.1007/s13244-018-0597-2 PMC5893493

[27] Cressoni M, Cadringher P, Colarieti A et al (2025) Is there a need for a CT scan of the pancreatic phase? A perfusion and simulation study of the pancreas, an HCC, and the kidney cortex. Rofo. 10.1055/a-2516-3176 10.1055/a-2516-3176 39914465

[28] Bae KT, Heiken JP, Brink JA (1998) Aortic and hepatic contrast medium enhancement at CT. Part II. Effect of reduced cardiac output in a porcine model. Radiology 207:657–662 9609887 10.1148/radiology.207.3.9609887

[29] Bae KT, Heiken JP, Brink JA (1998) Aortic and hepatic contrast medium enhancement at CT. Part I. Prediction with a computer model. Radiology 207:647–655 9609886 10.1148/radiology.207.3.9609886

[30] Awai K, Hiraishi K, Hori S (2004) Effect of contrast material injection duration and rate on aortic peak time and peak enhancement at dynamic CT involving injection protocol with dose tailored to patient weight. Radiology 230:142–150 14695390 10.1148/radiol.2301021008

[31] Remy-Jardin M, Remy J, Giraud F, Wattinne L, Gosselin B (1993) Computed tomography assessment of ground-glass opacity: semiology and significance. J Thorac Imaging 8:249–264 8246323 10.1097/00005382-199323000-00001

[32] Petersson J, Glenny RW (2014) Gas exchange and ventilation–perfusion relationships in the lung. Eur Respir J 44:1023–1041 25063240 10.1183/09031936.00037014

[33] Edwards RM, Godwin JD, Hippe DS, Kicska G (2016) A quantitative approach to distinguish pneumonia from atelectasis using computed tomography attenuation. J Comput Assist Tomogr 40:746–751 27560011 10.1097/RCT.0000000000000438

[34] Konietzke P, Steentoft HH, Wagner WL et al (2021) Consolidated lung on contrast-enhanced chest CT: the use of spectral-detector computed tomography parameters in differentiating atelectasis and pneumonia. Heliyon 7:e07066 34113729 10.1016/j.heliyon.2021.e07066 PMC8170158

[35] Grenier PA, Fetita CI, Brillet PY (2016) Quantitative computed tomography imaging of airway remodeling in severe asthma. Quant Imaging Med Surg 6:76–83 26981458 10.3978/j.issn.2223-4292.2016.02.08 PMC4775245

[36] Jobst BJ, Weinheimer O, Buschulte T et al (2019) Longitudinal airway remodeling in active and past smokers in a lung cancer screening population. Eur Radiol 29:2968–2980 30552475 10.1007/s00330-018-5890-4

[37] Washko GR, Dransfield MT, Estepar RS et al (2009) Airway wall attenuation: a biomarker of airway disease in subjects with COPD. J Appl Physiol (1985) 107:185–191 19407254 10.1152/japplphysiol.00216.2009 PMC2711787

[38] Higaki T, Nakaura T, Kidoh M et al (2018) Effect of contrast material injection duration on arterial enhancement at CT in patients with various cardiac indices: analysis using computer simulation. PLoS One 13:e0191347 29474457 10.1371/journal.pone.0191347 PMC5825030

[39] McFadyen JD, Stevens H, Peter K (2020) The emerging threat of (micro)thrombosis in COVID-19 and its therapeutic implications. Circ Res 127:571–587 32586214 10.1161/CIRCRESAHA.120.317447 PMC7386875

